# Supplementary material for: Population-scale peach genome analyses unravel selection patterns and biochemical basis underlying fruit flavor
Source: Nat Commun. 2021 Jun 14;12:3604. doi: 10.1038/s41467-021-23879-2 (PMC8203738; doi:10.1038/s41467-021-23879-2)
Supplement: Supplementary file 1 — Supplementary information [file 41467_2021_23879_MOESM1_ESM.pdf]

Population-scale peach genome analyses unravel selection patterns and biochemical basis underlying fruit flavor

Yu *et al.*

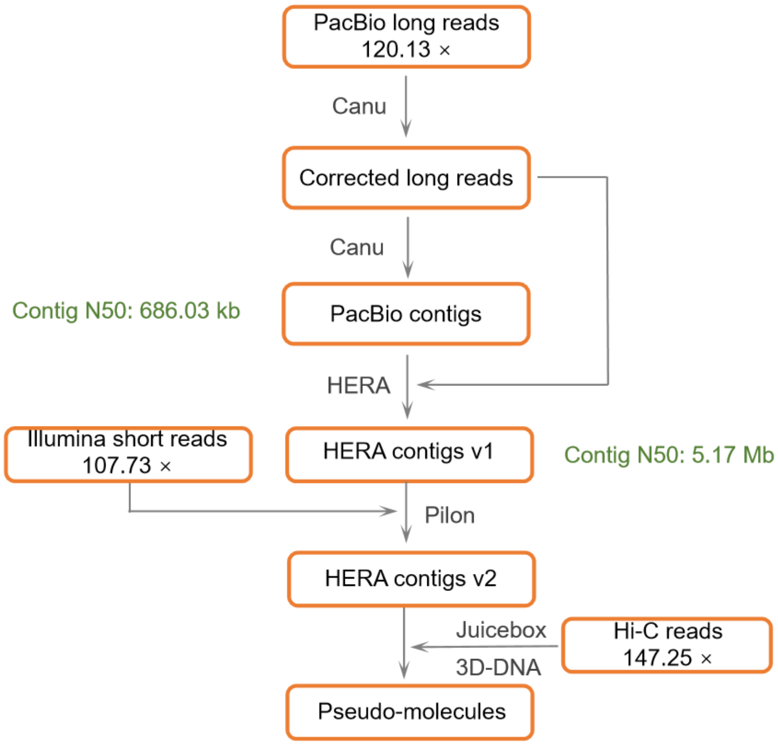

Supplementary Fig. 1. Overview of the pipeline used for the LHSM genome assembly. The LHSM PacBio long-reads were *de novo* assembled into PacBio contigs using Canu version 1.9. We then used the Highly Efficient Repeat Assembly (HERA) method based on the Canu-corrected PacBio long-reads to extend the PacBio contigs into contigs (HERA contigs v1). The Illumina short-read data was used for error correcting the contigs using Pilon. To anchor the corrected contigs (HERA contigs v2) into pseudo-chromosomes, the Hi-C sequencing data was first aligned into these contigs by Juicer v1.8.9 and, subsequently, the contigs were linked into 8 distinct pseudo-chromosomes.

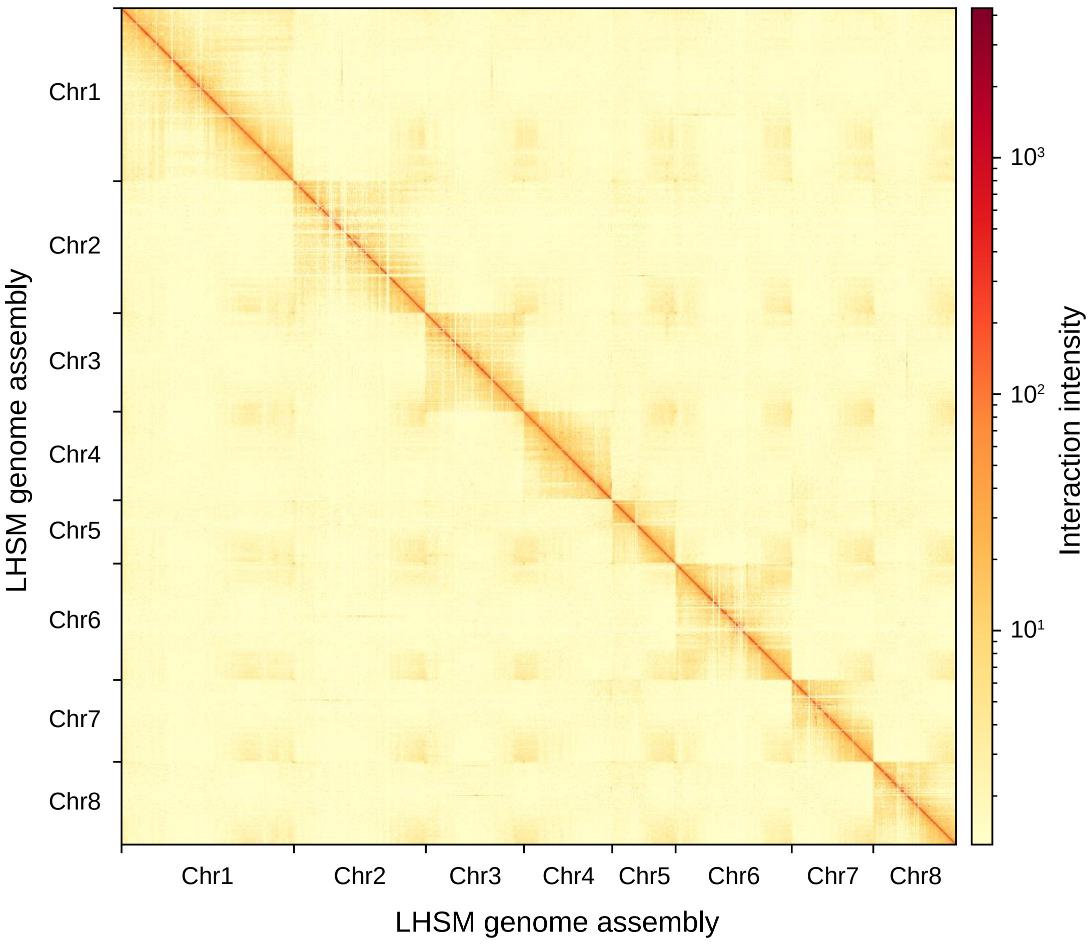

Supplementary Fig. 2. Genome-wide ICE-corrected Hi-C interaction heatmaps at 40-kb windows for the LHSM genome assembly. The diagonal lines represent the frequency of contact between two 40-kb loci on a chromosome.

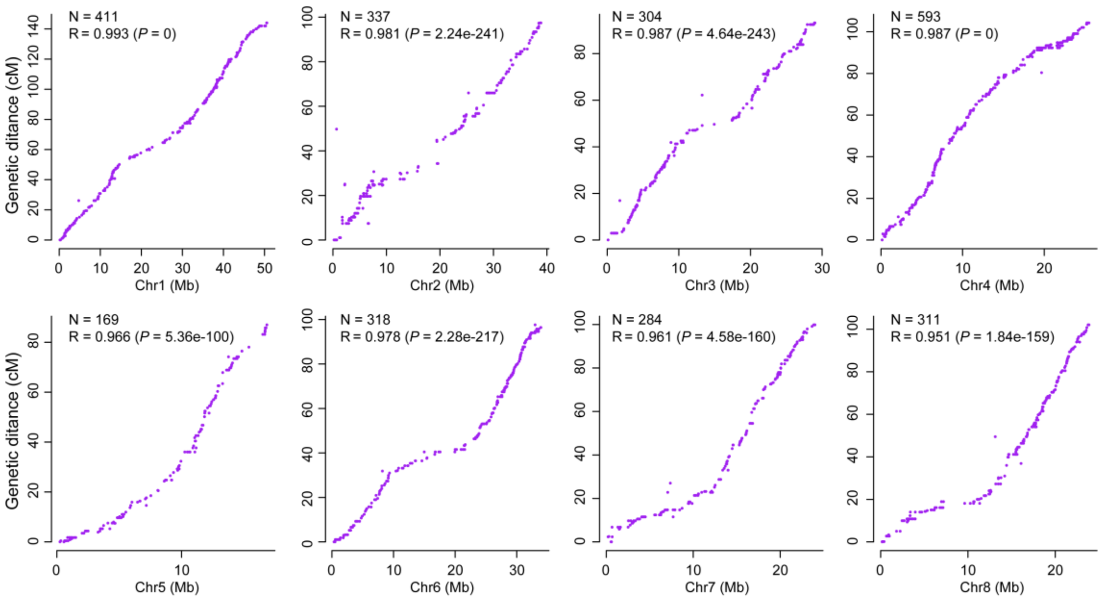

Supplementary Fig. 3. Collinear analysis of the peach genetic map and the LHSM genome assembly. The collinearity of the eight peach chromosomes and the linkage groups is shown in the different scatter plots. The X-axis represents the physical position of every marker on the chromosome. The Y-axis represents the genetic distance between the markers. N, number of markers; R, Pearson correlation coefficient (*P*-values in two-sided tests for each chromosome are shown). The genetic markers were obtained from a high-density multi-population consensus genetic linkage map[1]. Source data are provided as a Source Data file.

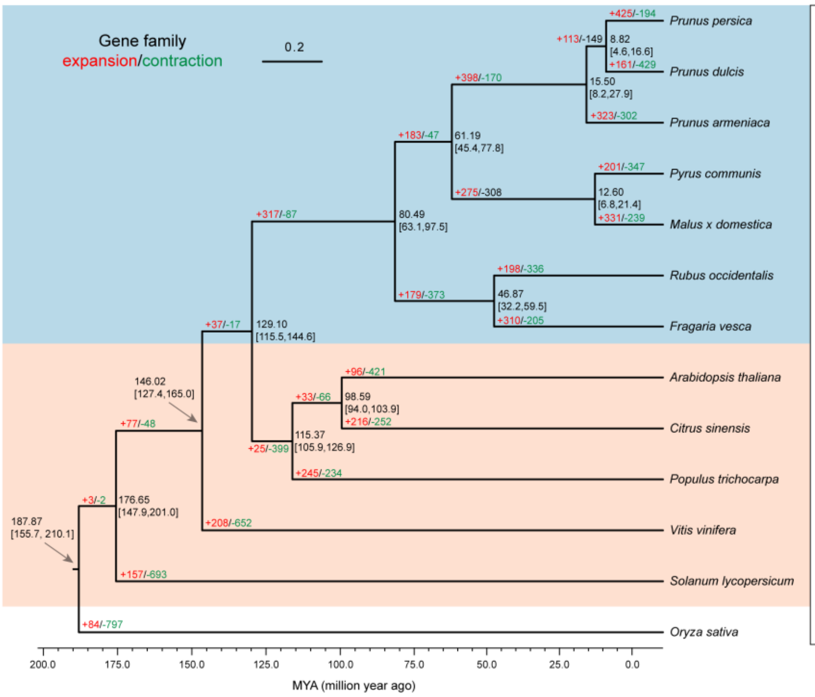

	Gene family	Specific family	Genes in family	Total genes
Prunus persica	16,561	595	33,208	35,215
Prunus dulcis	15,701	136	25,777	27,042
Prunus armeniaca	15,131	352	28,804	30,436
Pyrus communis	15,605	460	34,563	37,445
Malus x domestica	16,339	580	39,545	45,116
Rubus occidentalis	15,743	1,165	28,210	33,253
Fragaria vesca	15,643	935	29,966	33,538
Arabidopsis thaliana	13,250	734	24,813	27,416
Citrus sinensis	14,409	595	27,218	29,406
Populus trichocarpa	14,586	487	32,006	34,699
Vitis vinifera	15,127	773	27,161	31,845
Solanum lycopersicum	14,625	1,041	30,199	35,768
Oryza sativa	13,710	1,868	27,079	35,775

Supplementary Fig. 4. Genome evolution and gene family characteristics of *Prunus persica* (peach) and 11 other dicot plants using the monocot plant *Oryza sativa* (rice) as an out-group. This tree was generated using 367 single-copy ortholog families. The phylogenetic tree shown on the left shows the divergence time with 95% confidence intervals (CI) at each node. Light blue and orange backgrounds represent *Rosaceae* and non-*Rosaceae* species, respectively. The number of gene families, specific family, genes in the family and total number of genes are shown on the right for each species.

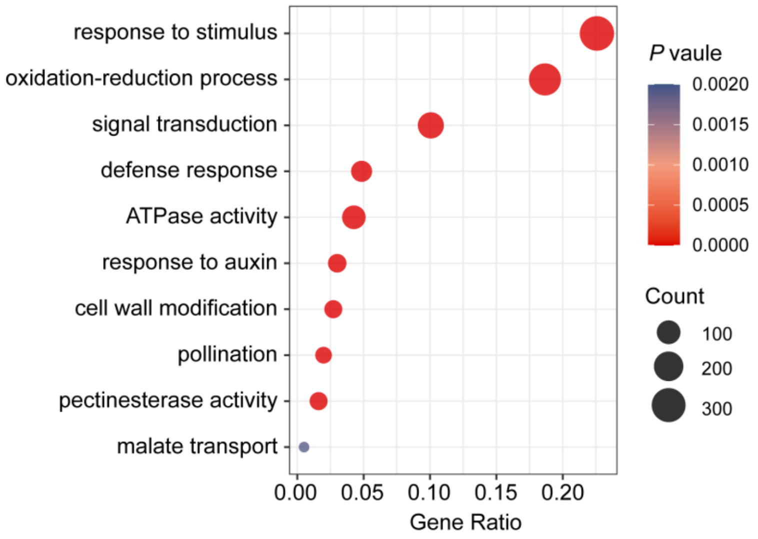

Supplementary Fig. 5. Significantly enriched GO terms of expanded gene families in *P. persica*. Top 10 enriched GO terms identified as significantly overrepresented (*P* value < 0.05, two-sided Fisher's exact test).

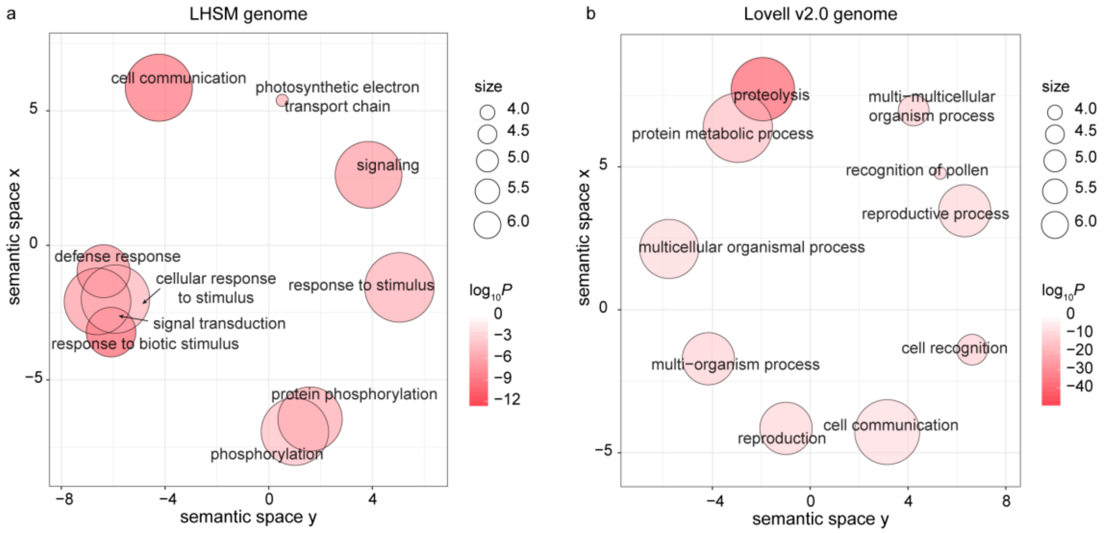

Supplementary Fig. 6. GO enrichment of the genes within the species-expanded orthogroups in the LHSM (a) and Lovell v2.0 (b) genomes. The GO terms displayed were ranked in the top 10 enriched biological processes. The color of the bubble indicates the *P* value (two-sided Fisher's exact test), whereas the size indicates the frequency of the GO terms.

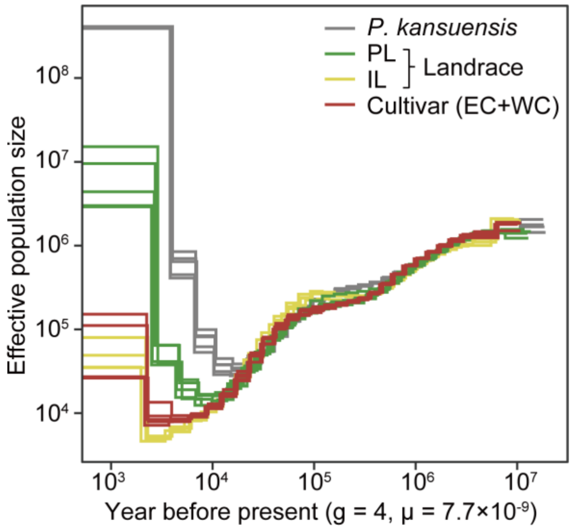

Supplementary Fig. 7. Multiple sequentially Markovian coalescent analysis for different groupings of peach accessions. The effective population fluctuations were inferred under a mutation rate $\mu = 7.7 \times 10^{-9}$ per site per generation and a generation time of 4 years.

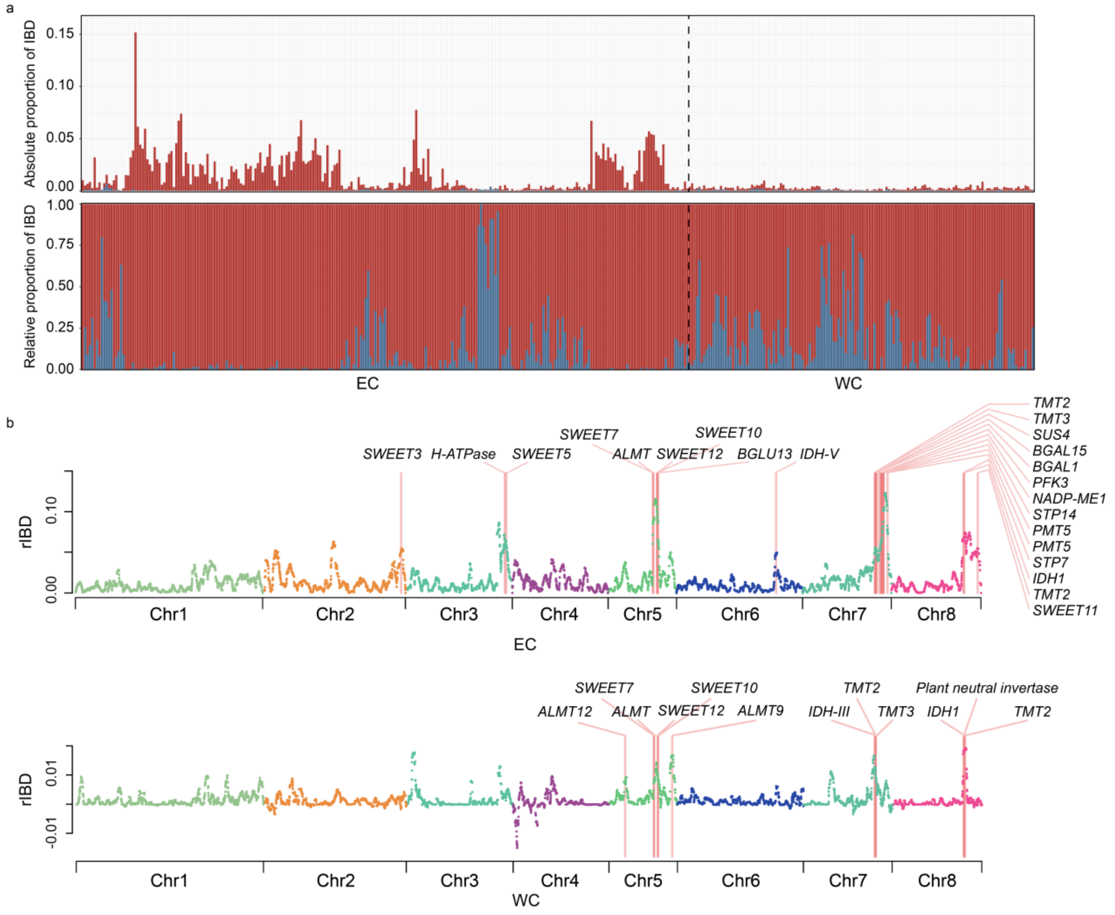

Supplementary Fig. 8. Genome-wide introgressions from PLs and ILs to modern peach cultivars (ECs and WCs). a, Introgression analysis for each of the modern peach cultivars, assessing introgression events from PLs or ILs based on rIBD analysis (upper panel). Stacked histograms for each cultivar represent the absolute proportion of introgressed genome segments from PLs (blue) or ILs (red). The lower panel shows the relative proportion of introgression from PLs (blue) versus ILs (red) for each modern peach cultivar. **b**, Genomic segments introgressed from ILs to ECs and WCs, respectively. The candidate gene within the introgressed segments that are associated with fruit flavor are labeled in red.

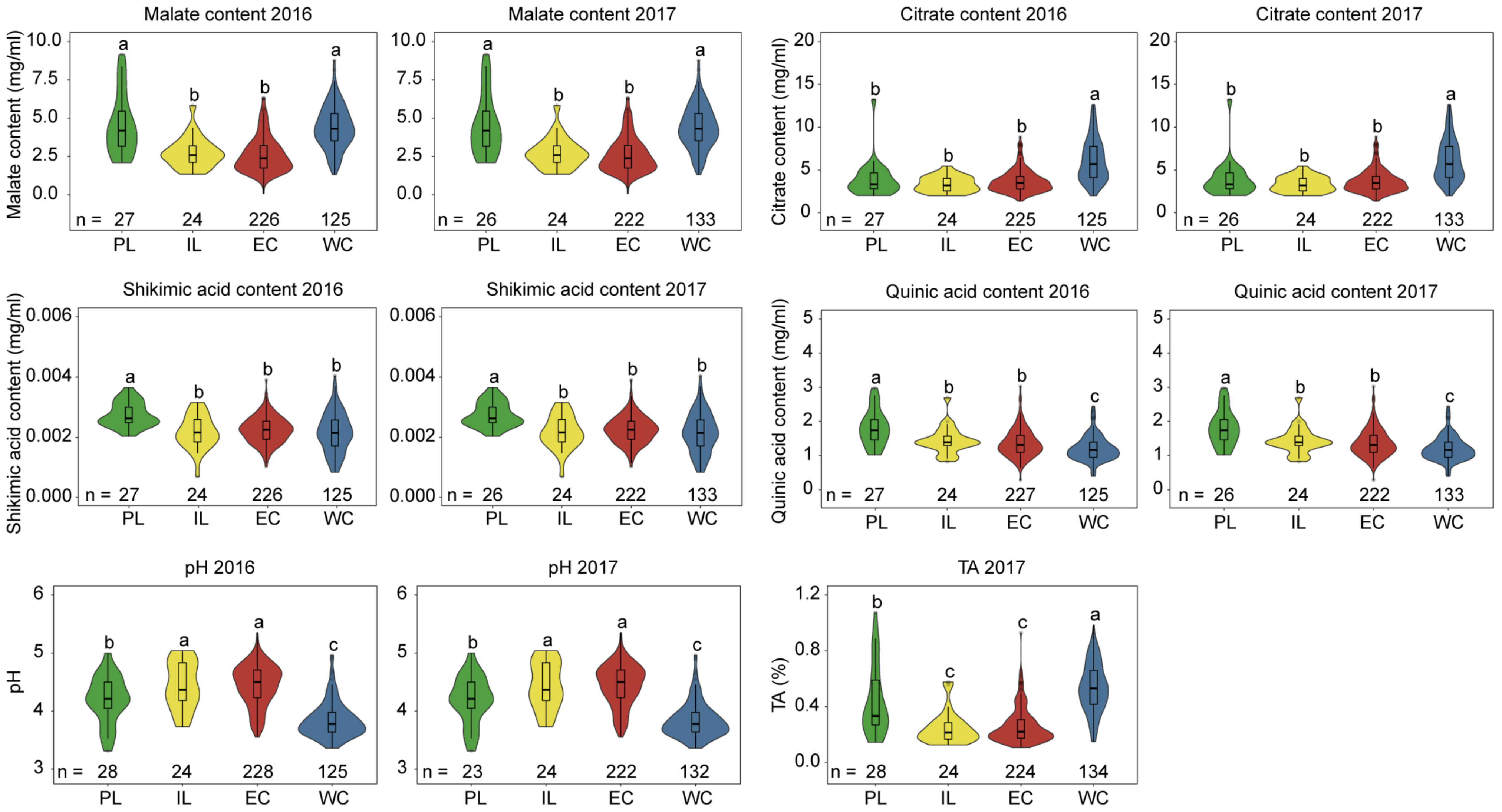

Supplementary Fig. 9. Fruit acidity-related phenotypes of different peach types. The fruit acidity-related phenotypes (pH, TA, and the content of malate, citrate, quinic acid and shikmic acid) for the PLs, ILs, ECs and WCs are shown in the violin plots from left to right, respectively. A multiple comparison analysis was conducted using the Least Significant Difference (LSD) test. The number of individuals for each peach type was shown below. In the violin plots, the central line represents the median values, the bounds of the box are correspond to the 25th and 75th percentiles, and the whiskers represent 1.5 * IQR (the interquartile range between the 25th and 75th percentiles).

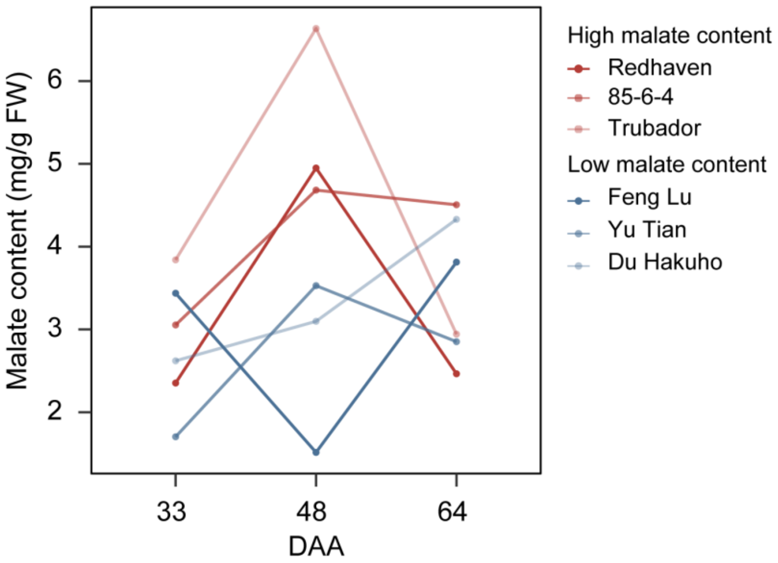

Supplementary Fig. 10. The accumulation curve of malate. The malate content of three high-malate WC accessions and three low-malate EC accessions at, respectively, 33, 48 and 64 days after anthesis (DAA). Source data are provided as a Source Data file.

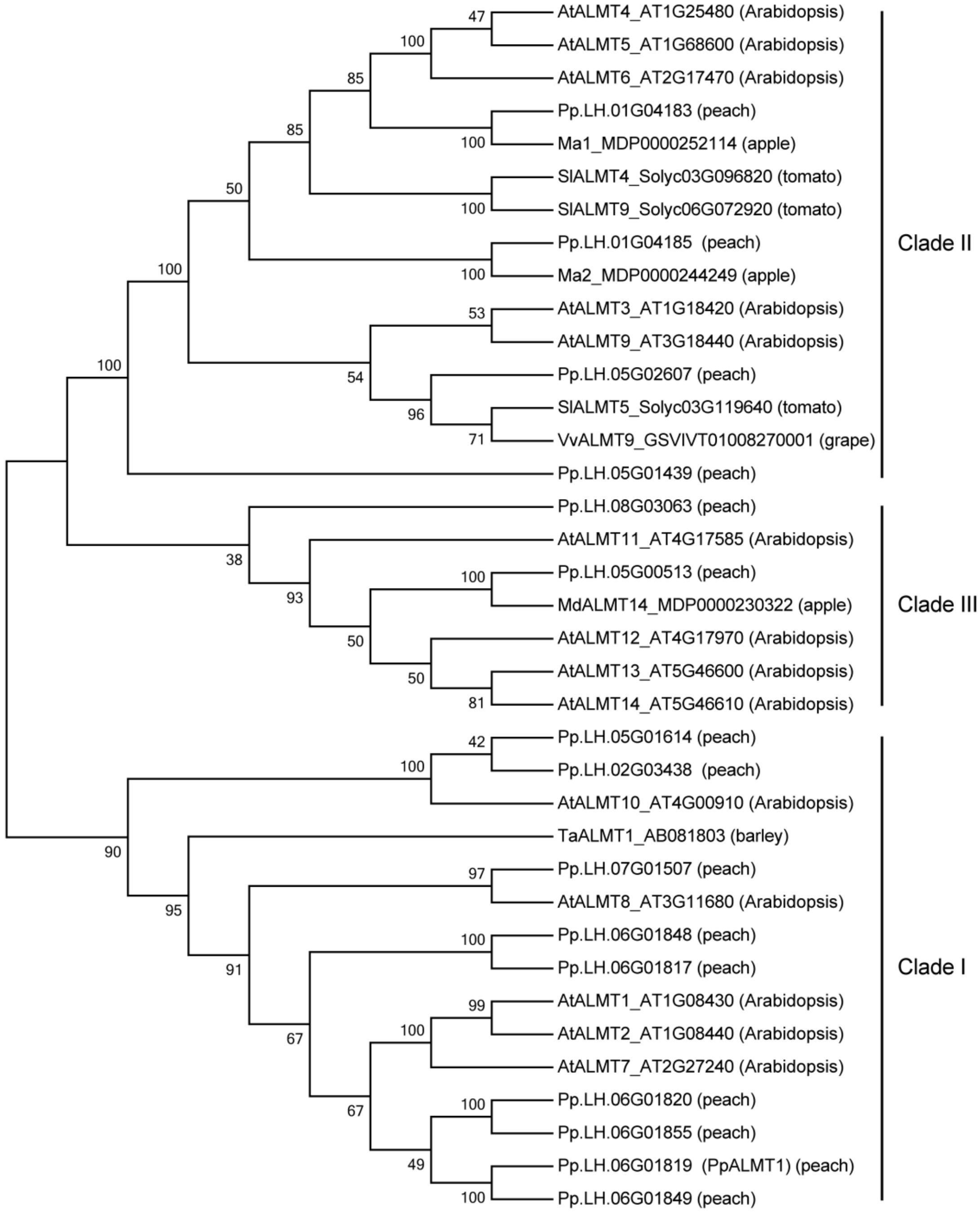

Supplementary Fig. 11. Phylogenetic tree for all 15 putative ALMT genes in the peach genome including PpALMT1 (Pp.LH.06G01819) and other previously reported ALMT proteins in Arabidopsis, apple, grapevine and barley. The clade classification was done in accordance to that of Arabidopsis[2]. The numbers at each node indicate the percentage of bootstrap support from 1,000 replicates.

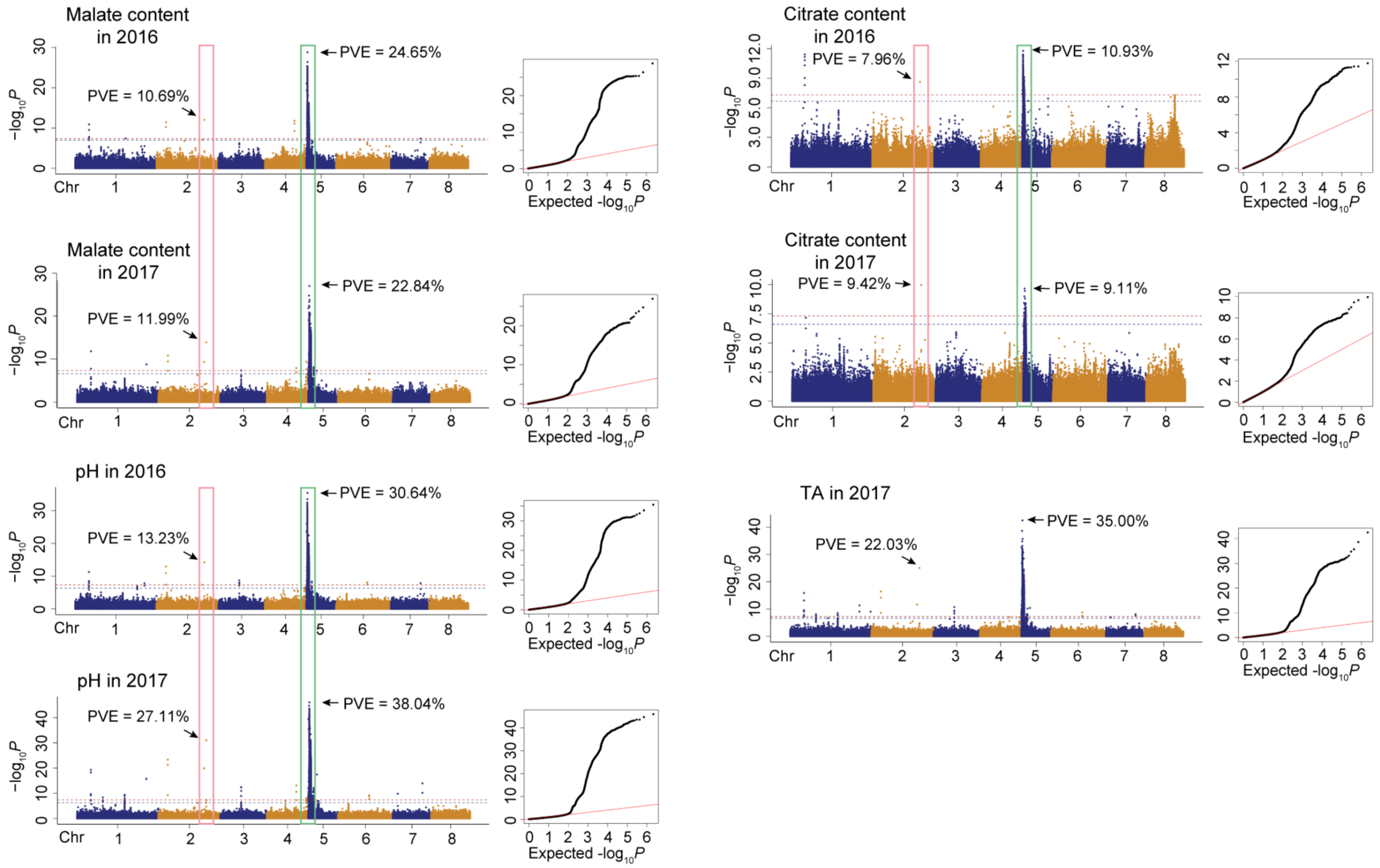

Supplementary Fig. 12.GWAS loci associated with pH, TA and the content of malate and citrate. The horizontal lines depict the Bonferroni-adjusted significance threshold (red) and Permutation threshold (blue) in the Manhattan plot. The PVE (phenotypic variance explanation) values of a strongly associated locus (Chr5: 21,714 – 1,812,811 bp) on chromosome 5 and a significantly associated locus (Chr2: 29,927,641 bp) on chromosome 2 are shown for their association with fruit acidity.

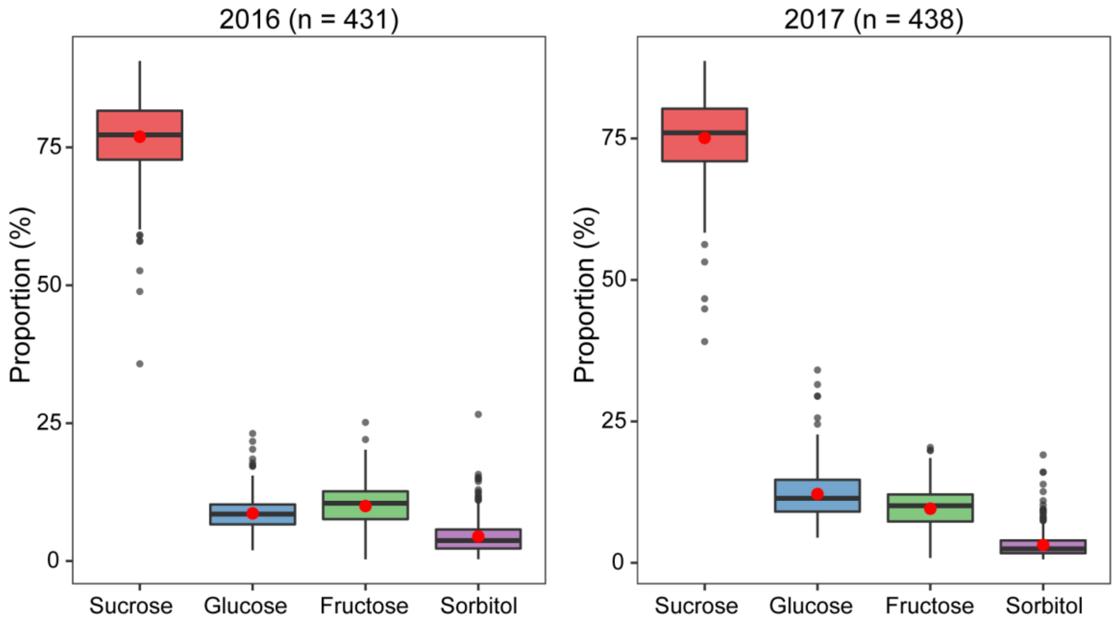

Supplementary Fig. 13. Relative proportions of four major sugars in each accession as measured in 2016 and 2017. The red dots represent average proportions. Numbers in parenthesis indicate number of individuals. In the box plots, the central line represents the median, the bounds of the box correspond to the 25th and 75th percentiles, and the whiskers represent 1.5 * IQR.

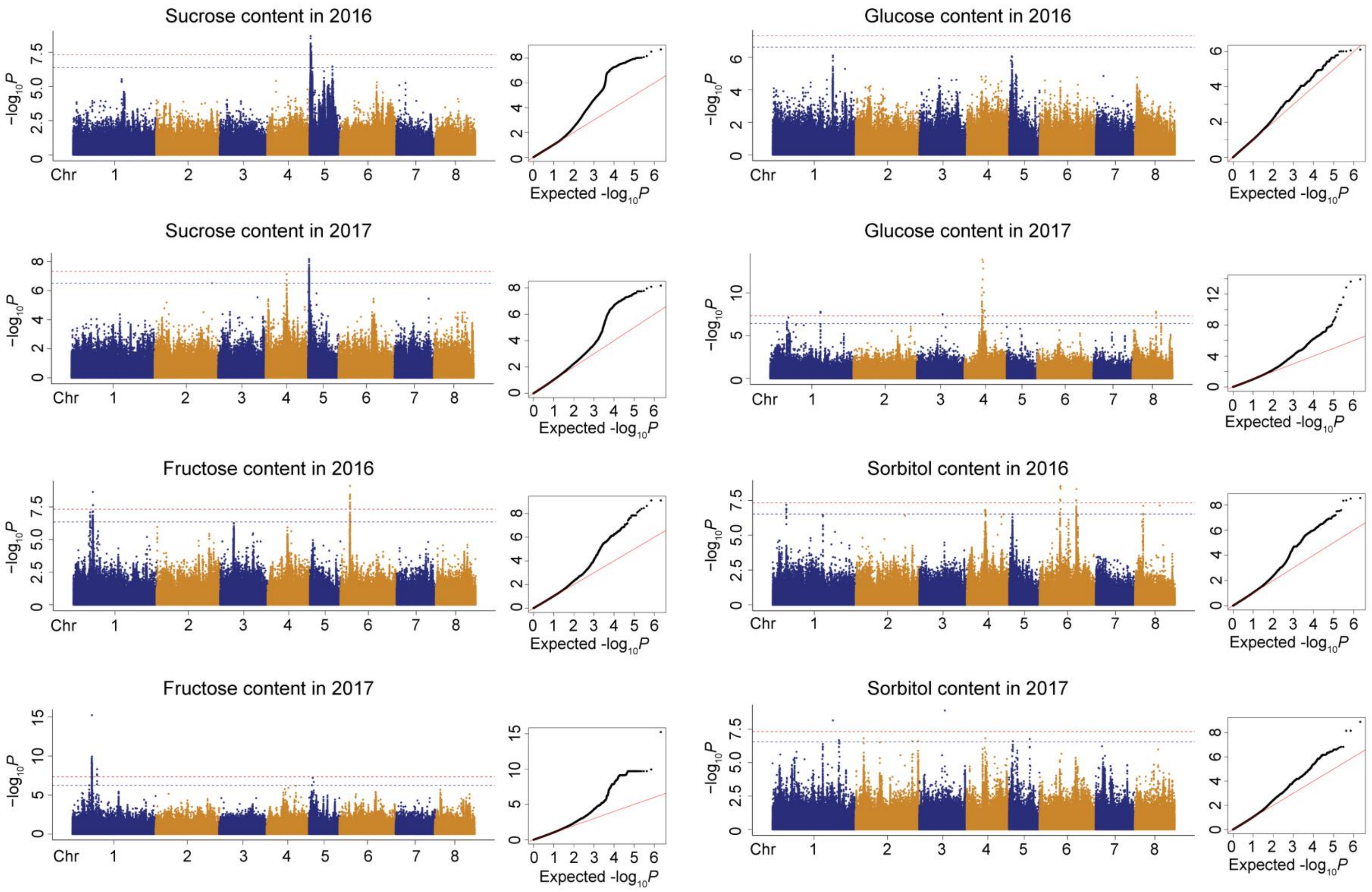

Supplementary Fig. 14. GWAS loci associated with the content of sucrose, glucose and fructose affecting peach fruit sweetness. GWAS results from analyses of data for sucrose, glucose fructose, and sorbitol content as measured in both 2016 and 2017. The horizontal lines depict the Bonferroni-adjusted significance threshold (red) and Permutation threshold (blue) in the Manhattan plot. The indicated PVE (phenotypic variance explanation) value represents the leading SNP at the major locus for each trait.

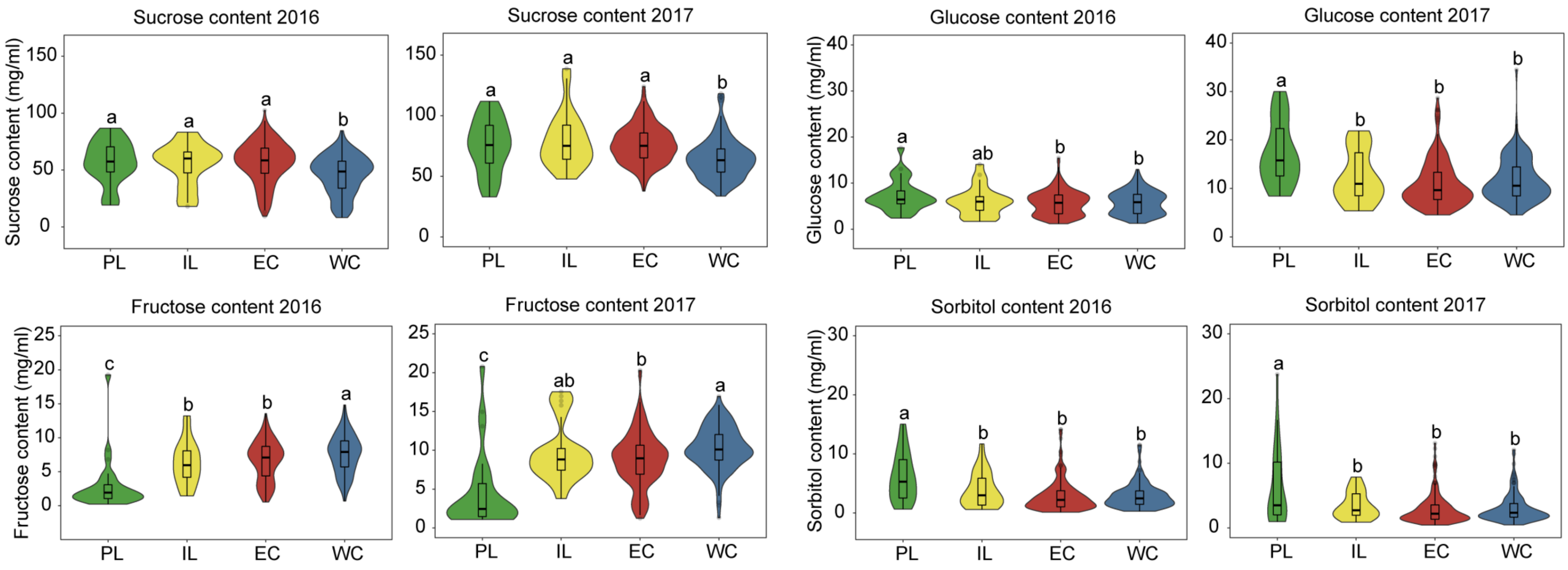

Supplementary Fig. 15. Major sugar-related phenotypes of different peach types. The major sugar-related phenotypes (sucrose, glucose, fructose and sorbitol) for the PLs, ILs, ECs and WCs are shown in violin plots. A multiple comparison analysis was conducted using the Least Significant Difference (LSD) test. In 2016, the sample numbers for PL, IL, EC, and WC are 27, 24, 226, and 125, respectively; in 2017, the sample numbers for PL, IL, EC, and WC are 26, 24, 222, and 133. In the violin plots, the central line represents the median, the bounds of the box correspond to the 25th and 75th percentiles, and the whiskers represent 1.5 * IQR.

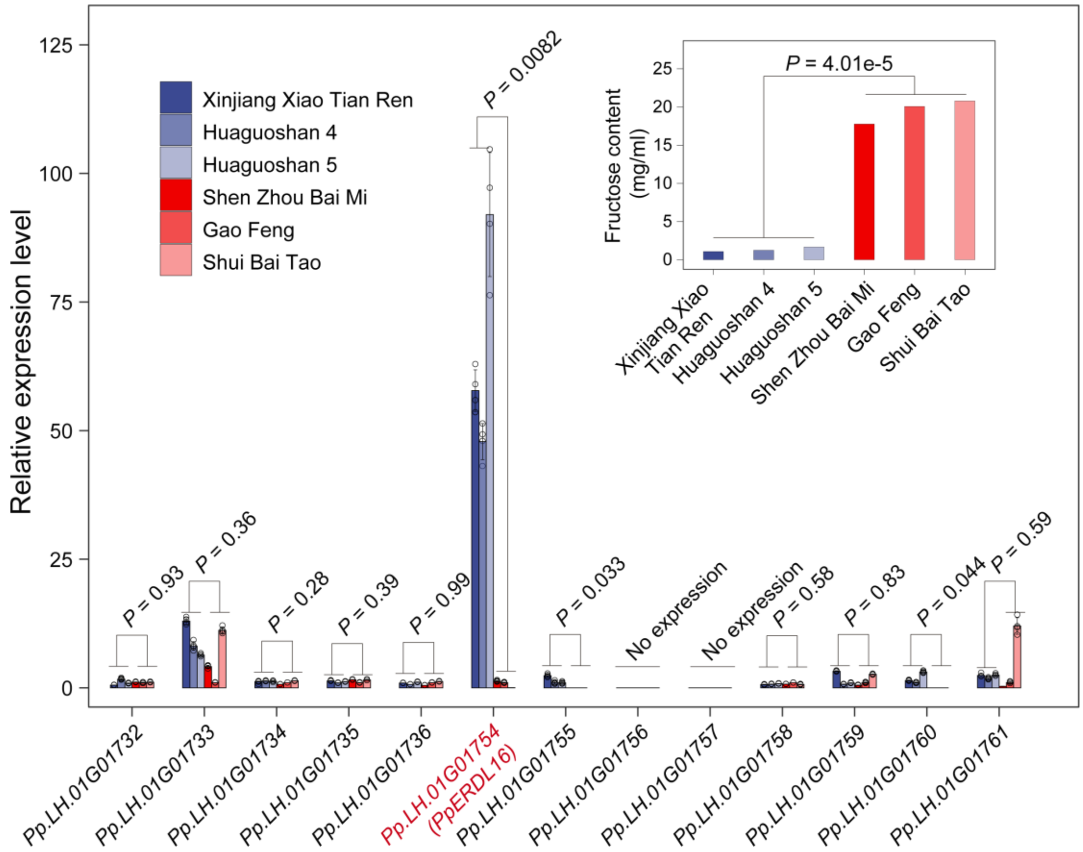

Supplementary Fig. 16. Relative expression levels for all 13 protein-coding genes within the two haplotype blocks. The relative expression levels of the 13 genes were compared between three low-fructose peach accessions and three high-fructose peach accessions. n = 4 independent experiments (mean ± SD, significance was tested with two-sided Student's *t*-tests). The fructose contents of these accessions were shown in the bar chart on the top right corner (Wilcoxon test). Source data are provided as a Source Data file.

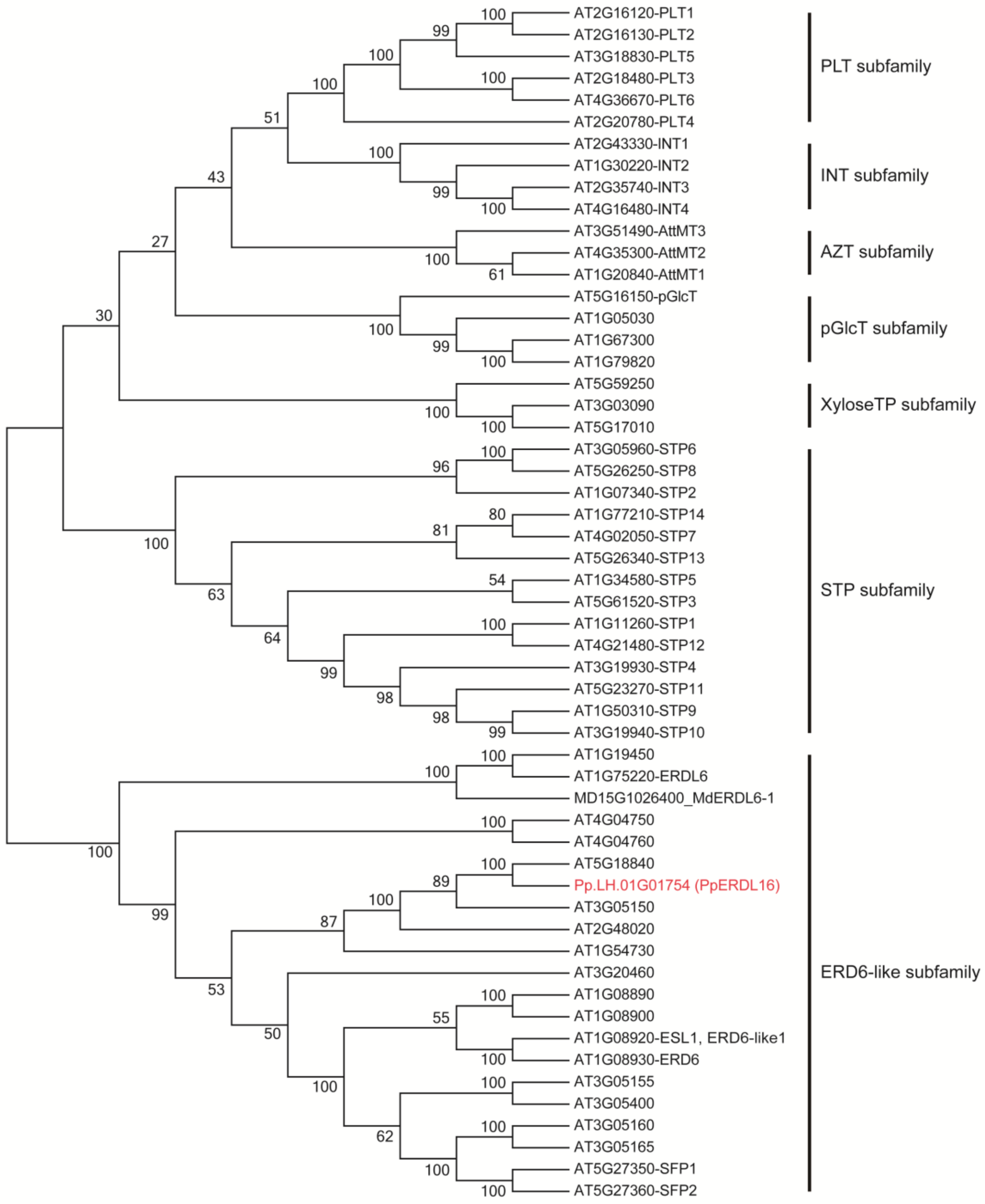

Supplementary Fig. 17. Phylogenetic tree for PpERDL16. The was built including 53 monosaccharide transporter (-like) (MST) proteins from all seven distinct subfamilies of Arabidopsis[3] and the recently reported MdERDL6-1 in apple[4] based on their complete protein sequences. The numbers at each node indicate the percentage of bootstrap support from 1,000 replicates. From the tree, it is possible to infer that PpERDL16 has the closest relationship with the AT5G1884 encoding sugar transporter ERD6-like 16 from the ERD6-like subfamily.

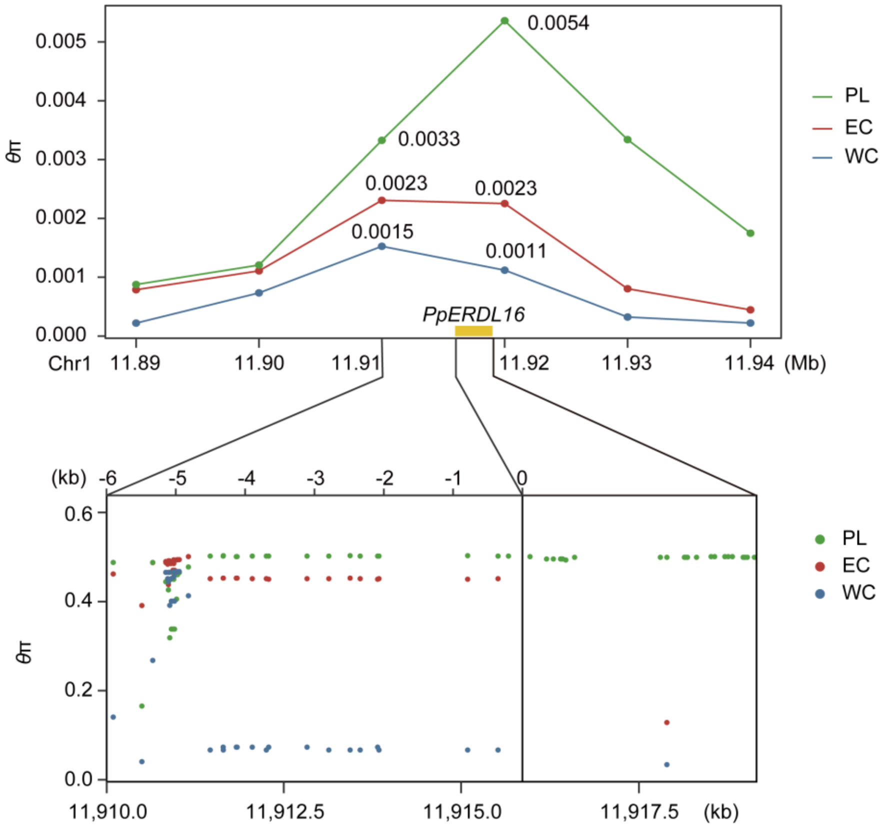

Supplementary Fig. 18. Reduced nucleotide diversity ($\theta\pi$) of *PpERDL16* during peach improvement. Displayed are the $\theta\pi$ values of consecutive windows (upper panel) and sites (lower panel) overlapping *PpERDL16*.

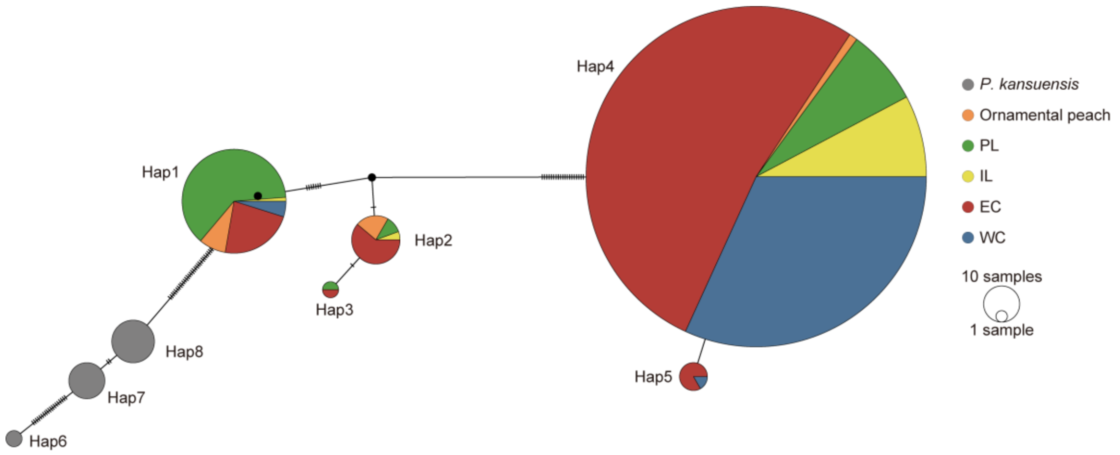

Supplementary Fig. 19. Haplotype network of *PpERDL16*. We used median-joint network analysis to construct the haplotype network of *PpERDL16* among all peach accessions (including 15 *P. kansuensis* accessions). The haplotypes that were present in a single accession were excluded. The node size and the parallel lines on branches represent the relative haplotype frequency and the number of mutations, respectively. The different colored portions in each node represent the proportion of different peach accessions within each haplotype.

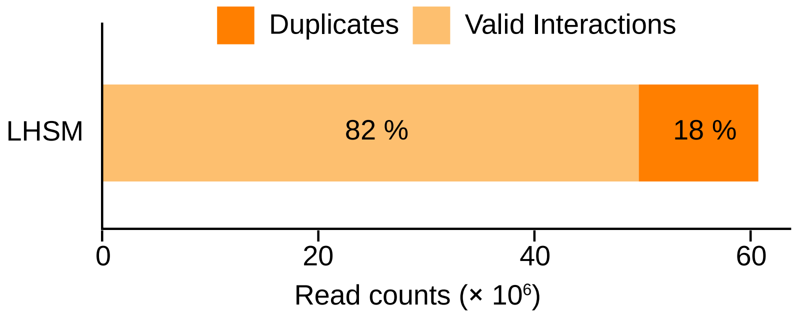

Supplementary Fig. 20. Mapping statistics of Hi-C interactions from the HiC-Pro results. The alignment of the Hi-C reads revealed a high proportion (82%) of valid interactions.

Supplementary Table 1. Information of Lovell and Longhua Shui Mi (LHSM).

Accession	Type	Organism	Fruit shape	Flesh color	Flesh taste	Hairiness	Adhesion	Texture	Flower	Leaf	Genome genotype
Lovell (PLov2-2N)	USA cultivar	*P. persica*	Round	Yellow	Acid	Peach	Freestone	Melting	Nonshowy	Red	Double haploid
LHSM	Chinese landrace	*P. persica*	Round	White	Low acid	Peach	Clingstone	Melting	Showy	Green	Diploid

Supplementary Table 2. Statistics of genome sequencing data for LHSM.

**Pacbio SMRT**	Read number	3,541,013
	Base nnumber	30,898,447,567
	N50 length	17 kb
	Depth	120.13 ×
**Illumina Novaseq**	Read number	184,730,160
	Base number	27,709,524,000
	Length	150
	Depth	107.73 ×
**Hi-C**	Read Pair number	126,246,024
	Base number	37,873,807,200
	Length	150
	Depth	147.25 ×

Supplementary Table 3. Genome survey of LHSM based on all Illumina reads.

**Genome survey**	**LHSM**
Estimated genome size	271 Mb
Estimated heterozygous ratio	0.32%
Estimated repeat ratio	54.26%

Supplementary Table 4. The length and gaps of chromosomes in LHSM genome assembly.

Chr	Length (bp)	Gaps
1	50,837,746	23
2	38,844,776	42
3	29,008,081	10
4	26,015,507	4
5	18,624,367	4
6	34,233,882	27
7	24,088,371	11
8	24,374,965	16
Total	246,027,695	137
**UnChr**	11,185,706	-
**Total**	257,213,401	-

Supplementary Table 5. Statistics of paired-end reads mapping.

**Reads**	Mapping rate (%)	98.63
	Coverage (%)	96.48
**Genome**	Coverage at least 4 × (%)	95.78
	Coverage at least 10 × (%)	95.05
	Coverage at least 20 × (%)	94.25

Supplementary Table 6. Assessment of LHSM genome assembly using expressed sequence tags (ESTs) from NCBI.

**ESTs**	**Number and percentage of aligned ESTs with different coverage**									**Not aligned ESTs**
	(80,100)	(70,80)	(60,70)	(50,60)	(40,50)	(30,40)	(20,30)	(10,20)	(0,10)	
80,805	76,042 (94.11%)	1,364 (1.69%)	763 (0.94%)	529 (0.65%)	283 (0.30%)	128 (0.16%)	56 (0.069%)	45 (0.057%)	32 (0.040%)	1,563 (1.93%)

Supplementary Table 7. Completeness of the LHSM and Lovell v2.0 genome assemblies.

Feature		Genome		Protein	
		LHSM	Lovell v2.0	LHSM	Lovell v2.0
BUSCOs	Complete BUSCOs	2,066 (97.4%)	2,054 (96.8%)	1,996 (94.1%)	1,986 (93.7%)
	Complete and single-copy BUSCOs	2,009 (94.7%)	2,016 (95.0%)	1,880 (88.6%)	1,904 (89.8%)
	Complete and duplicated BUSCOs	57 (2.7%)	38 (1.8%)	116 (5.5%)	82 (3.9%)
	Fragmented BUSCOs	13 (0.6%)	19 (0.9%)	73 (3.4%)	79 (3.7%)
	Missing BUSCOs	42 (2.0%)	48 (2.3%)	52 (2.5%)	56 (2.6%)
	Total	2,121	2,121	2,121	2,121
LAI		20.67	21.29	-	-

Supplementary Table 8. General statistics of predicted protein-coding genes and transcripts in the LHSM and Lovell v2.0 genome assemblies.

Feature	LHSM	Lovell v2.0
Gene number	35,215	31,972
Max gene length (bp)	35,934	36,290
Min gene length (bp)	156	156
Transcript number	40,072	47,089
Transcript number per gene**	1.0/1.1	1.0/1.2
Gene length (bp)*	1,490/2,175	1,536/2,215
mRNA length (bp)*	822/1,077	858/1,101
Max CDS length (bp)	15,525	15,615
CDS length (bp)*	819/1,075	855/1,100
Protein length (bp)*	272/357	284/366
Exon length (bp)*	150/255	150/256
Intron length (bp)*	167/338	165/336
5' UTR length (bp)*	100/82	100/81
3' UTR length (bp)*	216/331	234/287
Exon number*	2.0/4.3	2.0/4.3
Intron number*	1.0/3.2	1.0/3.3

* Means the median/average value.

** Means the median/average transcript number per gene.

Supplementary Table 9. Transcriptional data from different tissues for gene model prediction.

Samples	Tissues	NCBI SRA accessions
RRB19538	Flower	SAMN16191774
RRB19586	Fruit	SAMN16191775
RRB19635	Fruit	SAMN16191776
RRB19678	Fruit	SAMN16191777
SAMEA3861653	Root	SAMEA3861653
SAMEA3861654	Root	SAMEA3861654
SAMEA3861655	Root	SAMEA3861655
SAMEA3861656	Root	SAMEA3861656
SAMEA3861657	Root	SAMEA3861657
SAMEA3861658	Root	SAMEA3861658
SAMEA3861659	Leaves	SAMEA3861659
SAMEA3861660	Leaves	SAMEA3861660
SAMEA3861661	Leaves	SAMEA3861661
SAMEA3861662	Leaves	SAMEA3861662
SAMEA3861663	Leaves	SAMEA3861663
SAMEA3861664	Leaves	SAMEA3861664

Supplementary Table 10. Number and average percentage of TE-related genes.

Category	Total number
Total protein-coding gene	35,215
TE-related protein-coding genes	10,118
Average percentage of CDS overlapped by TEs	28.7%

Supplementary Table 11. Statistics of gene annotations for LHSM genome.

Type	Number	Percent (%)
Pfam	22,278	63.26
InterPro	23,301	66.17
NR	31,009	88.06
GO	17,123	48.62
KEGG	6,658	18.91
Annotated	31,092	88.29
Unannotated	4,123	11.71
Total	35,215	100

Supplementary Table 12. Statistics of repeat sequences for LHSM and Lovell v2.0 genomes.

Class	Subclass	LHSM			Lovell v2.0*		
		Number	Length (bp)	Percent	Number	Length (bp)	Percent
Retrotransposons	**Total**	82,851	53,119,243	20.65%	74,168	45,685,587	20.09%
	LTR elements	73,444	50,490,963	19.63%	65,807	43,511,756	19.13%
	Gypsy	29,447	23,800,350	9.25%	25,444	20,370,864	8.96%
	Copia	32,578	22,590,100	8.78%	31,847	19,929,250	8.76%
	Others	11,419	4,100,513	1.59%	8,516	3,211,642	1.41%
	Non-LTR elements	9,407	2,628,280	1.02%	8,361	2,173,831	0.96%
	SINEs	3,352	490,885	0.19%	3,346	332,625	0.15%
	LINEs	6,055	2,137,395	0.83%	5,015	1,841,206	0.81%
DNA transposons	**Total**	67,677	34,841,912	13.55%	54,462	27,835,940	12.24%
	CMC-EnSpm	11,186	16,295,231	6.34%	10,337	12,764,703	5.61%
	MULE-MuDR	11,349	4,789,507	1.86%	10,926	4,244,278	1.87%
	PIF-Harbinger	12,679	4,973,317	1.93%	9,387	4,048,348	1.78%
	hAT-Ac	7,139	2,069,871	0.80%	5,299	1,563,235	0.69%
	Helitron	5,028	1,791,088	0.70%	2,671	1,090,852	0.48%
	Others	20,296	4,922,898	1.91%	15,842	4,124,524	1.81%
Other repeats	**Total**	200,220	30,393,725	11.82%	185,258	27,121,234	11.93%
	Unkown	106,877	26,257,595	10.21%	96,492	23,331,215	10.26%
	Satellites	164	74,770	0.03%	36	14,546	0.01%
	Simple repeats	78,520	2,962,654	1.15%	74,553	2,761,202	1.21%
	Low complexity	13,854	666,725	0.26%	13,495	647,657	0.28%
	Others	805	431,981	0.17%	682	366,614	0.16%
**Total**		350,748	118,354,880	46.01%	313,888	100,642,761	44.26%

*Re-annotated repeat sequences using the same pipeline as used for the LHSM genome.

Supplementary Table 13. Number of SNPs and InDels between the LHSM and Lovell v2.0 genomes.

Chr	SNPs	InDels
**1**	91,148	30,436
**2**	129,517	31,639
**3**	76,232	18,723
**4**	132,882	23,955
**5**	47,380	11,599
**6**	70,426	22,450
**7**	58,266	16,568
**8**	60,319	18,161
**UnChr**	39,709	8,257
**Total**	705,879	181,788

Supplementary Table 14. Number of affected gene by SNPs and InDels between the LHSM and Lovell v2.0 genomes.

Large-effect mutations	Number of mutations		Number of affected genes		
	SNPs	InDels	SNPs	InDels	Total
Nonsynonymous substitutions	27,156	-	8,077	-	-
Frameshift deletions	-	2,017	-	1,124	-
Frameshift insertions	-	2,172	-	1,199	-
Stop gain	686	145	564	130	-
Stop loss	197	25	190	25	-
Splicing	225	90	205	84	-
**Total**	28,264	4,449	8,212	2,022	10,234

Supplementary Table 15. Summary of InDels and rearranged regions detected between the LHSM and Lovell v2.0 genomes.

	Number	Total size (bp)	Largest size (bp)	Smallest size (bp)	Median size (bp)
**InDels in syntenic regions**					
Deletions	2,653	2,244,272	57,622	51	196
Insertions	2,068	2,166,363	22,533	51	222
**Rearranged regions**					
Duplications	1,320	8,596,275	271,863	202	3,383
Inversions	45	6,099,144	1,267,002	213	8,754
Translocations	391	11,217,468	922,691	233	6,339

Supplementary Table 16. The synteny alignment between the structured rearranged region of LHSM and scaffold of the Lovell v2.0 genome.

Code	LHSM		Lovell v2.0		
	Chromosome	Position on chromosome	Scaffold	Position on scaffold	Putative mis-orientation or mis-ordering
I	Chr3	13,312,855–13,870,436	Super_32	575,509–45,224	mis-orientation
II	Chr3	14,334,748–15,228,781	Super_18	973,342–659	mis-orientation
III	Chr3	15,230,244–16,451,437	Super_18	2,092,361–1,008,570	mis-orientation
IV-1	Chr3	16,628,662–17,336,800	Super_27	122,428–786,082	mis-ordering
IV-2	Chr3	17,337,291–18,863,662	Super_451	3–1,445,406	mis-ordering

Supplementary Table 17. Analysis of orthologous genes of the LHSM and Lovell v2.0 genomes.

Type	LHSM	Lovell v2.0
Number of genes	35,215	31,972
Number (percentage) of genes in orthogroups	34,198 (97%)	31,153 (97%)
Number (percentage) of unassigned genes	1,017 (3%)	819 (3%)
Number (percentage) of orthogroups containing species	25,732 (100%)	25,633 (99%)
Number (percentage) of species-conserved orthogroups	22,166 (63%)	22,166 (69%)
Number of genes in species-conserved orthogroups	23,726	23,726
Number of species-expanded orthogroups	2,419	944
Number (percentage) of genes in species-expanded orthogroups	7,727 (22%)	2,988 (9%)
Number of species-specific orthogroups	203	104
Number (percentage) of genes in species-specific orthogroups	1,303 (4%)	590 (2%)

Supplementary Table 18. The threshold of GWAS based on permutation test.

Trait	Year	Threshold
Citrate	2016	6.68
Citrate	2017	6.59
TA	2017	6.64
Malate	2016	6.97
Malate	2017	6.60
pH	2016	6.31
pH	2017	6.26
Fructose	2016	6.35
Fructose	2017	6.24
Glucose	2016	6.63
Glucose	2017	6.42
Sorbitol	2016	6.53
Sorbitol	2017	6.55
Sucrose	2016	6.39
Sucrose	2017	6.51

Supplementary Table 19. Number and percentage of GWAS loci positioned within or nearby selective regions between ECs and WCs.

Trait	Number of GWAS signals	Number of peak SNPs with overlap or nearby selective sweep	Percentage
Citrate2016	4	4	100.00%
Citrate2017	3	2	66.67%
Malate2016	7	3	42.86%
Malate2017	10	3	30.00%
pH2016	10	3	30.00%
pH2017	19	7	36.84%
TA2017	15	4	26.67%

Supplementary Table 20. Primers used in this study.

Primer ID	Primer sequences (5'-3')	Purpose
qACT	Forward: GTTATTCTTCATCGGCGTCTTCG	qRT-PCR for gene *Pp.LH.06G02290* (*PpACT*)
	Reverse: CTTCACCATTCCAGTTCCATTGTC	
q227	Forward: GCTAACAACATTGAGAAACTTGGGA	qRT-PCR for gene *Pp.LH.06G01819* (*PpALMT1*)
	Reverse: TCTTCACTCTGTTTTGAGTTGAGAA	
q541	Forward: CGGCCCAAGGAATATGAAGAAC	qRT-PCR for gene *Pp.LH.01G04183* (Putative *PpALMT*)
	Reverse: CATTGTCGCATGGAAGGATCTG	
q542	Forward: TGGTCAACTCAGAGAGGTGGGA	qRT-PCR for gene *Pp.LH.01G04185* (*Putative PpALMT*)
	Reverse: CCTCAGGTCAAGCACAGTTTCA	
q902	Forward: CACAGATTGATCTATCGTAACCTGG	qRT-PCR for gene *Pp.LH.02G03438* (*Putative PpALMT*)
	Reverse: CACACATTTATACCCTTTGATTTCC	
q900	Forward: TCTCAGATCTTTTCCTGTGCCA	qRT-PCR for gene *Pp.LH.05G02607* (*Putative PpALMT*)
	Reverse: CCTGTTTCATGAACATGCTTCC	
q162	Forward: GAGTGCTGTCAAGGCTCTACCGT	qRT-PCR for gene *Pp.LH.01G01761*
	Reverse: GACTTGGAAGGTTTGGTTCAGAT	
q163	Forward: CTGGTTCTTCTGGTCCTCTTCTAC	qRT-PCR for gene *Pp.LH.01G01758*
	Reverse: CCACCGATTTCTGAGCTTCC	
q164	Forward: TGCTTCTTCAGCCTGGTAGAG	qRT-PCR for gene *Pp.LH.01G01757*
	Reverse: GGCACAAGTGCCCACTGGAC	
q165	Forward: TTCGCCACCTTCTATCCCCTAT	qRT-PCR for gene *Pp.LH.01G01756*
	Reverse: GCTATTATCTTTTCCAAACCTCGT	
q166	Forward: TGCCTTATCTTGCTTTTGGGTGTA	qRT-PCR for gene *Pp.LH.01G01755*
	Reverse: TTCTTGAATTTCGGTCATTTCATC	

Supplementary Table 20 (continued)

Primer ID	Primer sequences (5'-3')	Purpose
q167	Forward: CTCCTGTTTTTCCTTCTTTTTGT	qRT-PCR for gene *Pp.LH.01G01754* (*PpERDL16)*
	Reverse: GGGTGTTATTTTCTCCATTTTCA	
q169	Forward: GTTGTTTTCGTTTATTTGTTCGCTC	qRT-PCR for gene *Pp.LH.01G01759*
	Reverse: TAAATGAACCCTACCATCACCACCT	
q170	Forward: TTCAATTATGTGTGATCGTTAGTGG	qRT-PCR for gene *Pp.LH.01G01760*
	Reverse: ATCCTTGTAAGTTGTGAGGGTTGTT	
q684	Forward: TAACAGAAGATAGAAGCATGACCAC	qRT-PCR for gene *Pp.LH.01G01736*
	Reverse: ACCATACAAACATAAAAGAGAGGGA	
q685	Forward: TGAGGCAGTGGCATCAACTAAGG	qRT-PCR for gene *Pp.LH.01G01734*
	Reverse: CAAGCAAACTTCTGAATTTTGGG	
q686	Forward: CCACCTGCACTGATTATATTGTCTC	qRT-PCR for gene *Pp.LH.01G01733*
	Reverse: TCAAAGATTGTGAACTTTGTTCCC	
q720	Forward: CTATCACAGTTGTTCTTTTCAGCA	qRT-PCR for gene *Pp.LH.01G01735*
	Reverse: ATTGATTTAGGACTAGATGGTTCG	
q721	Forward: GGAAAGACTCCAATCCAAGTTTACC	qRT-PCR for gene *Pp.LH.01G01732*
	Reverse: GATGAGAAGGATATTGAAGCTCACC	
ALMT1	Forward: GGAATTCCGTTTCAAAGCTGAAGAACATCAA	Gene cloning for gene *PpALMT1* used in transient expression analysis
	Reverse: CAAGCTTGAAGGTGGCGACAAGTTCAGG	
PF1	Forward: CGGAATTCCGGAGTCCTACCAGGGGCAGAG	Gene cloning for gene *PpERDL16* used in transient expression analysis
	Reverse: CCAAGCTTGGTATCAGCTAATGATGTCTTGTCTTC	
PF2	Forward: CCTTAATTAAATGGCAATTGGGCAGTTCAG	*PpERDL16* gene cloning for pMD85-*PpERDL16-GFP* vector
	Reverse: TTGGCGCGCCAGCTAATGATGTCTTGTCTTCTTGAT	construction used in subcellular localization analysis

Supplementary References

1. da Silva Linge, C. *et al*. High-density multi-population consensus genetic linkage map for peach. *PloS ONE* **13**, e0207724 (2018).
2. Kovermann, P. *et al*. The Arabidopsis vacuolar malate channel is a member of the ALMT family. *Plant J*. **52**, 1169–1180 (2007).
3. Johnson, D. A., Hill, J. P. & Thomas, M. A. The monosaccharide transporter gene family in land plants is ancient and shows differential subfamily expression and expansion across lineages. *BMC Evol. Biol*. **6**, 1–20 (2006).
4. Zhu, L. *et al*. MdERDL6-mediated glucose efflux to the cytosol promotes sugar accumulation in the vacuole through up-regulating TSTs in apple and tomato. *P. Natl. Acad. Sci*. USA. **118**, e2022788118 (2021).
