# Supplementary material for: Population-scale peach genome analyses unravel selection patterns and biochemical basis underlying fruit flavor
Source: Nat Commun. 2021 Jun 14;12:3604. doi: 10.1038/s41467-021-23879-2 (PMC8203738; doi:10.1038/s41467-021-23879-2)
Supplement: Supplementary file 4 — Description of additional supplementary files [file 41467_2021_23879_MOESM4_ESM.docx]

**Description of Additional Supplementary Files**

Title: Supplementary Data 1

Description: Information of expansion/contraction gene families in the evolutional analysis of *Prunus persica* (peach) and the other 11 dicot plants.

Title: Supplementary Data 2

Description: PAV regions between the LHSM and Lovell v2.0 genomes.

Title: Supplementary Data 3

Description: Information of LHSM-specific and Lovell-specific PAV genes.

Title: Supplementary Data 4

Description: Information of InDels in syntenic regions and rearranged regions between the LHSM and Lovell v2.0 genomes.

Title: Supplementary Data 5

Description: Information of protein-coding genes located within or overlapped with the InDels and rearranged regions.

Title: Supplementary Data 6

Description: Summary statistics of sequencing data for each accession analyzed in this study.

Title: Supplementary Data 7

Description: Distribution of SNPs in different genomic regions for each accession.

Title: Supplementary Data 8

Description: Distribution of InDels in different genomic regions for each accession.

Title: Supplementary Data 9

Description: Protein-coding genes within the selective sweep regions in the comparison of ILs vs. PLs.

Title: Supplementary Data 10

Description: Significantly enriched GO terms for genes within the selective sweep regions in the comparison of ILs vs. PLs.

Title: Supplementary Data 11

Description: Putatively introgressed segments from ILs to ECs using rIBD analysis.

Title: Supplementary Data 12

Description: Protein-coding genes within the putatively introgressed segments from ILs to ECs.

Title: Supplementary Data 13

Description: Putatively introgressed segments from ILs to WCs using rIBD analysis.

Title: Supplementary Data 14

Description: Protein-coding genes within the putatively introgressed segments from ILs to WCs.

Title: Supplementary Data 15

Description: Significantly enriched GO terms of protein-coding genes within the selective sweep regions in the comparison of ECs vs. WCs.

Title: Supplementary Data 16

Description: Significantly enriched GO terms of protein-coding genes within the selective sweep regions in the comparison of WCs vs. ECs.

Title: Supplementary Data 17

Description: Possible genes under selection by comparing of ECs vs. WCs.

Title: Supplementary Data 18

Description: Possible genes under selection by comparing of WCs vs. ECs.

Title: Supplementary Data 19

Description: Phenotypic correlation of fruit acidity related traits.

Title: Supplementary Data 20

Description: Significantly associated loci and phenotypic variance explanation (PVE) of the peak SNPs for acidity-related traits.

Title: Supplementary Data 21

Description: QTLs associated with sugar content related traits in peach.
