# Supplementary material for: Population-scale peach genome analyses unravel selection patterns and biochemical basis underlying fruit flavor
Source: Nat Commun. 2021 Jun 14;12:3604. doi: 10.1038/s41467-021-23879-2 (PMC8203738; doi:10.1038/s41467-021-23879-2)
Supplement: Supplementary file 5 — Reporting Summary [file 41467_2021_23879_MOESM5_ESM.pdf]

nature research

Corresponding author(s): Hua Xie, Jianhua Wei, Quan Jiang

Last updated by author(s): 2021/May/12

Reporting Summary

Nature Research wishes to improve the reproducibility of the work that we publish. This form provides structure for consistency and transparency in reporting. For further information on Nature Research policies, see our Editorial Policies and the Editorial Policy Checklist.

Statistics

For all statistical analyses, confirm that the following items are present in the figure legend, table legend, main text, or Methods section.

n/a	Confirmed	
☐	☒	The exact sample size (*n*) for each experimental group/condition, given as a discrete number and unit of measurement
☐	☒	A statement on whether measurements were taken from distinct samples or whether the same sample was measured repeatedly
☐	☒	The statistical test(s) used AND whether they are one- or two-sided<br>*Only common tests should be described solely by name; describe more complex techniques in the Methods section.*
☒	☐	A description of all covariates tested
☐	☒	A description of any assumptions or corrections, such as tests of normality and adjustment for multiple comparisons
☐	☒	A full description of the statistical parameters including central tendency (e.g. means) or other basic estimates (e.g. regression coefficient) AND variation (e.g. standard deviation) or associated estimates of uncertainty (e.g. confidence intervals)
☐	☒	For null hypothesis testing, the test statistic (e.g. F, t, r) with confidence intervals, effect sizes, degrees of freedom and P value noted<br>*Give P values as exact values whenever suitable.*
☒	☐	For Bayesian analysis, information on the choice of priors and Markov chain Monte Carlo settings
☒	☐	For hierarchical and complex designs, identification of the appropriate level for tests and full reporting of outcomes
☐	☒	Estimates of effect sizes (e.g. Cohen's *d*, Pearson's *r*), indicating how they were calculated

Our web collection on statistics for biologists contains articles on many of the points above.

Software and code

Policy information about availability of computer code

Data collection	No software was used in data collection.
Data analysis	We only used freely software available informatics for our data analysis.<br>Canu (v1.9), Pilon (v1.23), Juicer (v1.8.9), RepeatMasker (v4.1.0), RepeatModeler (v2.0), LTR_FINDER (v1.07), AUGUSTUS (v3.2), MAKER (v3.01.03), HISAT (v2.1.0), StringTie (v2.1.0), InterProScan (v5.27-66.0), BLAST (v2.5.0+), BWA-MEM (v0.7.17-r1188), BUSCO (v3.0.2), GMAP (v2019-09-12), OrthoFinder (v2.3.9), RAxML (v8.2.12), PAML (v4.9), CAFE (v4.2), MUMmer (v4.0.0beta2), Minimap2 (v2.17-r941), fastp (v0.20.1), Picard (v1.118), GATK (v4.1.5.0), FastTree2 (v2.1.10), ADMIXTURE (v1.3.0), smartPCA (v16000), Beagle (v5.1), RefinedIBD (v17Jan20.102), MSMC2 (v2.1.1), PopLDdecay (v3.41), VCFtools (v0.1.17), XPCLR (v1.0), Selscan (v1.1.0), ClusterProfiler (v3.18.0), Fast-LMM (v2.06.20130802), Plink (v1.90b6.10), PopART (v1.7.1)

For manuscripts utilizing custom algorithms or software that are central to the research but not yet described in published literature, software must be made available to editors and reviewers. We strongly encourage code deposition in a community repository (e.g. GitHub). See the Nature Research guidelines for submitting code & software for further information.

Data

Policy information about availability of data

All manuscripts must include a data availability statement. This statement should provide the following information, where applicable:
- Accession codes, unique identifiers, or web links for publicly available datasets
- A list of figures that have associated raw data
- A description of any restrictions on data availability

Data supporting the findings of this work are available within the paper and its Supplementary Information files. A reporting summary for this Article is available as a Supplementary Information file. The datasets and plant materials generated and analyzed during the current study are available from the corresponding author

upon request. The raw resequencing data have been deposited in the Sequence Read Archive (https://www.ncbi.nlm.nih.gov/sra/) of the National Center for Biotechnology Information (NCBI) under BioProjects PRJNA715782 and PRJNA663114. The genome assembly has been deposited at GenBank under the accession JAGEPH000000000. The raw PacBio data and Hi-C data are available in the NCBI Sequence Read Archive under BioProject PRJNA707388. Source data are provided with this paper.
Pfam: http://pfam.xfam.org/.
InterPro: https://www.ebi.ac.uk/interpro.
NR: https://www.ncbi.nlm.nih.gov/refseq/about/nonredundantproteins/.
GO: http://geneontology.org.
KEGG: https://www.genome.jp/kegg/.

Field-specific reporting

Please select the one below that is the best fit for your research. If you are not sure, read the appropriate sections before making your selection.

☒ Life sciences ☐ Behavioural & social sciences ☐ Ecological, evolutionary & environmental sciences

For a reference copy of the document with all sections, see nature.com/documents/nr-reporting-summary-flat.pdf

Life sciences study design

All studies must disclose on these points even when the disclosure is negative.

Sample size	We collected resequencing data for 564 peach accessions (548 P. persica genomes from various geographic regions and 15 closely wild relative (P. kansuensis) genomes). These included 379 newly sequenced accessions from this study and 185 accessions from previous studies. The sample size was sufficient for the GWAS analysis.
Data exclusions	No data were excluded from analysis.
Replication	All experiments in the study have been repeated successfully at least three times.
Randomization	For phenotypic data collection, the measured samples were randomly selected. The accessions were randomly selected for the expression level measurement of the candidate genes.
Blinding	Investigators were blinded at phenotypic data collection. For all experimental analysis, investigators were blinded.

Reporting for specific materials, systems and methods

We require information from authors about some types of materials, experimental systems and methods used in many studies. Here, indicate whether each material, system or method listed is relevant to your study. If you are not sure if a list item applies to your research, read the appropriate section before selecting a response.

Materials & experimental systems

n/a	Involved in the study
☒	☐ Antibodies
☒	☐ Eukaryotic cell lines
☒	☐ Palaeontology and archaeology
☒	☐ Animals and other organisms
☒	☐ Human research participants
☒	☐ Clinical data
☒	☐ Dual use research of concern

Methods

n/a	Involved in the study
☒	☐ ChIP-seq
☒	☐ Flow cytometry
☒	☐ MRI-based neuroimaging
